# The Impact of Global Institutions on National Health HIV/AIDS Policy Making in Developing Countries

**DOI:** 10.5195/cajgh.2013.27

**Published:** 2013-09-06

**Authors:** Zhanat Mokushev

**Affiliations:** London School of Economics and Political Sciences

**Keywords:** HIV/AIDS Policy Making, WTO, IMF, World Bank

## Abstract

This article explores the relationship of global institutions such as the International Monetary Fund, World Trade Organization, World Bank, and individual developing countries in social health policy making in terms of HIV and AIDS. We examine the role of IGOs and NGOs in regarding to HIV/AIDS issues then analyse the TRIPs agreement as a tool for developing countries to negotiate with International organisations in global health policy decisions.

## Introduction

One of the most significant, current discussions around the world is the impact of various forms of globalisation. It is obvious that globalisation affects migration patterns, national economies, and cultural and political developments.^1^ In recent years, there has been an increasing interest in the role of the International Monetary Fund (IMF), World Bank, and World Trade Organization (WTO).^2^ However, these rapid changes are having serious positive and negative effects on national health policymaking. For example, questions have been raised about the social policy of individual countries under the Global Health Policy in terms of HIV and AIDS treatment.^3^ Many authors contend that Global Health Policy dictates their rules and conditions to individual countries,^4^ but there has been little discussion about the role of states in the process of social health policy priority decision-making in relation to global institutions. As a result, while some claim that individual countries still decide their own Trade-Related Intellectual Property (TRIPs) social health policy priorities, others regard it as the domination of global social policy making, particularly in HIV/AIDS.^5^

The aim of this article is to examine the possibilities of individual developing countries in making decisions about their own social policy priorities in terms of HIV and AIDS treatment. We first give a brief overview of the impact of globalisation, then examine the role of different global institutions such as the IMF, WTO and World Bank regarding to HIV/AIDS issues. Next, we analysed the capabilities of individual developing countries in decision making of their own social policies in terms of TRIPs agreement. Finally, we assess the place of developing countries in global health policy decisions.

## Global institutions: understanding their divergent impact on policy making

The role of global institutions “as important policy actors is widely recognized in relation to the social policy processes for developing countries”.^6^ Multilateral organisations like the IMF, World Bank, and WTO became more powerful in global policy making than other bilateral or individual countries.^7^ It is important to focus on understanding their divergent impact on domestic policy making.

In the past three decades, “IMF intervention in domestic policy making has increased commensurately”.^7^ On the one hand, the IMF can support national economies through the prolonged use of resources by recipient countries. On the other hand, “the views expressed regarding the impact of prolonged use on the policy formulation process were generally negative”.^7^ According to Rowden, many poor “countries are under pressure to stay ‘on track’ with their IMF programs”. For example, most developing countries cannot increase funding to fight against HIV/AIDS because they are “under current IMF policy choices and spending constraints”.^8^

Furthermore, other global institutions like the WTO have intruded into national policy, and “have extended its authority into areas of domestic regulation, legislation, governance and policy making central to the development process”.^7^ Other contend that in spite of the strong restrictions and rules of the WTO, in some cases the WTO uses the principle “one country, one vote”.^4^ As a result, it gives some developing countries opportunity to use this principle in its national policy making. Nevertheless, though there are many compromises and negotiations between global institutions and individual countries, the former “still remain embedded in local decision making”.^7^ Many authors maintain that the main objective of the WTO “is to regulate and facilitate world trade [and] it is not a welfare-oriented organization”.^9^

In the era of globalisation, the role of global institutions is obvious. However over the past two decades, non-governmental organisations and institutions have started to play significant roles in policy decision making, particularly in health policy.^8^ For example, the World Health Organisation (WHO) proclaims that every human have rights to the highest healthcare standard.^9^ There were many programmes providing health policy in the past by WHO such as the Declaration of Alma Ata (1978), Primary Health Care (1979) and others, which were a main agenda to the strategy of “Health for All”.^9^ It is also established as the central objective of international and national health activities by the nation states throughout the world.^9^

In spite of the fact that the main objective of WHO is providing healthcare for humanity, many scholars maintain that “the World Bank is the greatest single donor in health and one of the greatest single donors in the fight against HIV/AIDS”.^9^ Owing to the structural adjustment policy, the World Bank could influence domestic health policy making.^10^ In 1993, the World Bank published the World Development Report (WDR), which focused on health issues.^11^ It is clear that the main document of the World Bank “had an important conceptual influence on health system reforms in the 1990s”; however, many researchers argue “the Bank tried to link an expansion of social services to neoliberal economic concepts”.^9^

As a result, international organizations and institutions started to provide the Global Social Policy in terms of health. Developed countries recognize diseases like HIV/AIDS in developing countries on a global scale. Consequently, “health is increasingly perceived as a global public good that requires strengthened global efforts”.^9^ Nevertheless, these issues lead to look more deeply to health problems by developed countries and significantly increased their interest to the HIV/AIDS diseases.

It has been argued that “the self-interest of rich countries may be one of the most important driving forces behind” global health governance.^9^ For example, in 2002 the Global Fund was established, which mainly targeted the fight against HIV/AIDS in developing countries.^9^ The Global Fund included a great number of different global institutions and developed countries (like the World Bank and G8 countries) as well as recipient countries (like India and Brazil). Besides, the developing states “create a Country Coordinating Mechanism with the participation of all stakeholders (including civil society and private sector) that is authorized to apply for funds to conduct programmes”.^9^ As a result, in this case, individual countries still can protect their own social policy priorities through efforts such as the Global Fund.

While there was a growing role of new policy making actors, it was inevitable to escape “conflicts around the TRIPS agreement and the access to treatment for millions of HIV/AIDS patients”.^9^

## The role of individual countries in social policy priorities: the issue of TRIPs agreement

An increasing number of scholars suggest that Global Social Policy national countries cannot totally decide their own social policy priorities.^12^ However, despite the fact that many countries integrated into the Global Social Policy, many authors assert that individual countries still can decide their own social policy priorities partially, particularly in the case of TRIPs.^13^ Contrary to this belief, many researchers contend that new actors’ participation in global health policy making led to barriers and conflicts like “the access to medicines under the conditions of the internationalization of intellectual property rights through TRIPS” in the South.^9^

According to Correa,^14^ “under the TRIPS Agreement, all WTO Member countries became bound to grant patents for pharmaceutical products”, particularly for HIV/AIDS drugs. This secures investment into innovation and protects against free riders but introducing a patent system in some countries imposes a social cost.^15^ While some claim that these HIV/AIDS treatments were available, others argue that it did not satisfy the needs of poor countries.^9^ Thus, the concern was that TRIPS serves the interests of major producers of pharmaceutical products and restricts access to essential medicine for the poor. As Correa notes: “developing countries were coerced to accept the new standards set forth by the agreement in exchange for the benefits they would supposedly obtain in other areas, such as agriculture and textiles”.^14^

Nevertheless, some developing countries might provide compulsory licensing provisions in respect to TRIPs’ rights to produce drugs, especially for treating HIV/AIDS. Generic drug manufacturers in India, China, and Brazil are challenging the monopoly of the drug transnationals.^7^ Such patent regime allowed the Indian pharmaceutical industry to thrive. It makes India one of the most efficient manufacturers of generic medicine, giving this country’s substantial expertise in reverse engineering and a new method of producing pharmaceutical goods. The same pattern can be seen in other developing countries like Brazil.^16^ Overall, the opportunity to design the patent regime that meets the particular needs of each country increased the world supply of low-cost, generic medicines; poor, developing countries benefited from this.

However, most drugs manufacturers argued that compulsory licenses did not permit trade in generic medicine in accordance TRIPs.^13^ Correa asserts that in the 1990s, Thailand’s government tried to produce specific HIV/AIDS drugs like ddI, invented by pharmaceutical manufacturer “Bristol-Myers Squibb” in the USA; in turn, the US government imposed trade sanctions on exports to Thailand.^14^ As a result, the massive production of drugs in Thailand decreased dramatically.

Developing countries tried to find compromise with developed countries to use compulsory licenses- they promised not to produce medicines in case wealthy countries decreased the price of drugs, particularly for poor people.^5^ Stiglitz suggests that rich countries cannot play “one-size-fits-all” policies. The granting of a compulsory license may be an important tool to introduce competition and thereby lower the prices and affordability drugs”, but developing countries were right to demand a TRIPs’ revision.^5^ In order to cope with HIV/AIDS problems, in 2001 Brazil forced the US pharmaceutical companies to decrease the prices of medications for the treatment of HIV/AIDS by threatening them with compulsory licences and parallel importation.^15^ As a result, it was estimated that a generic medicine would become available for more than 600,000 HIV-positive patients in the country.^5^ Therefore, to some extent the limitation to patent right in TRIPS could be effective to pursue public interests.

Another problem was that some developing countries (like in Sub-Sahara Africa) could not afford to set up production of essential medicine under the TRIPs compulsory licensing arrangements and therefore failed to secure their domestic health situation.^16^ They also could not import cheap medicine from other countries because export and import of patented goods under compulsory licensing initially was not permitted by TRIPs.^5^ Many critics accuse the TRIPS Agreement of limiting the poor’s access to essential generic medicine, worsening the AIDS crisis.^9^ In order to address such a failure, the WTO Ministerial Conference adopted the “Doha Declaration” on the TRIPS Agreement in 2001 and Waiver Decision in 2003.^5^,^15^,^16^ Stiglitz, for example, suggests that we “waive” the tax allowing [poor countries] to use the intellectual property for their own citizens”.

Some developing countries were satisfied; they expressed concerns about the complexity of its arrangements.^14^ The USA proposed that the system must be applied to limited lists of diseases agreed by WTO Members.^16^ Even so, the USA proposal was denied and is now under the discretion of the individual member to decide whether the public health situation needs to be addressed through granting mandatory licences to the exporters of pharmaceutical products.^16^ Critics have also argued “the developing countries are simply free-riding on the advanced industrial countries”.^5^

It is clear that individual countries can decide their own social policy priorities. TRIPs illustrates that through cooperation among developing countries, negotiation and mitigation with global institutions and organizations can be successful. On the contrary, many authors argue that the Waiver Decision does not establish a straightforward and expeditious system (Abbott et al, 2007). The procedure of granting compulsory licences for exports contains many bureaucratic formalities that discourage the wide use of this system.^16^ Thus, according to evidence, it seems reasonable to conclude that individual countries can still partially decide their own social policy priorities.

## Conclusions: the place of individual countries in Global Health policy making

Over the past three decades, the role of global institutions (IMF, WTO and the World Bank) has increased rapidly. Also, in the 1990s “due to the domination of economic globalization concepts, there was slow progress in implementation of human rights to making decision in health by developing countries” (Kohlmorgen, 2008:99).

Secondly, there is an increasing role of health diseases like HIV/AIDS on a global scale, allowing developing countries (like India and Brazil) to use their power in domestic making decision. Nevertheless, patents for producing HIV/AIDS drugs have two sides that are affecting developed and developing countries. They foster the development of new drugs that contribute to health care and wealth creation in developed countries; however, patents impede broad access to such drugs in developing countries, while they fail to promote the development of drugs needed by the poor”.^14^

Finally, implicated is the possibility that in the era of globalization, individual countries can provide their own social health policy due to the TRIPS Agreement (like compulsory licenses, parallel imports), though in some cases the developed countries try to create barriers.
